# DNA Methylation Profiles Are Stable in H3 K27M-Mutant Diffuse Midline Glioma Neurosphere Cell Lines

**DOI:** 10.3390/children11040492

**Published:** 2024-04-20

**Authors:** Matthew J. Schniederjan, Cahil Potnis, Varshini Vasudevaraja, Catherine D. Moser, Bethany Watson, Matija Snuderl, Tobey MacDonald, Beverly B. Rogers

**Affiliations:** 1 Department of Pathology, Children’s Healthcare of Atlanta, Atlanta, GA 30342, USAbethany.watson@choa.org (B.W.);; 2 Department of Pathology, Emory University School of Medicine, Atlanta, GA 30322, USA; 3 Department of Biomedical Informatics, New York University Langone Health, New York, NY 10016, USA; varshini.vasudevaraja@nyulangone.org; 4 Department of Neuropathology, New York University Langone Health, New York, NY 10016, USA; 5 Department of Pediatrics, Aflac Cancer and Blood Disorders Center, Children’s Healthcare of Atlanta, Emory University School of Medicine, Atlanta, GA 30322, USA

**Keywords:** DNA methylation, H3 K27M, cell culture, diffuse midline glioma, DIPG

## Abstract

Diffuse midline gliomas are among the deadliest human cancers and have had little progress in treatment in the last 50 years. Cell cultures of these tumors have been developed recently, but the degree to which such cultures retain the characteristics of the source tumors is unknown. DNA methylation profiling offers a powerful tool to look at genome-wide epigenetic changes that are biologically meaningful and can help assess the similarity of cultured tumor cells to their in vivo progenitors. Paraffinized diagnostic tissue from three diffuse intrinsic pontine gliomas with H3 K27M mutations was compared with subsequent passages of neurosphere cell cultures from those tumors. Each cell line was passaged 3–4 times and analyzed with DNA methylation arrays and standard algorithms that provided a comparison of diagnostic classification and cluster analysis. All samples tested maintained high classifier scores and clustered within the reference group of H3 K27M-mutant diffuse midline gliomas. There was a gain of 1q in all cell lines, with two cell lines initially manifesting the gain of 1q only during culture. In vitro cell cultures of H3 K27M-mutant gliomas maintain high degrees of similarity in DNA methylation profiles to their source tumor, confirming their fidelity even with some chromosomal changes.

## 1. Introduction

Diffuse midline gliomas (DMGs), which most commonly occur in children as diffuse intrinsic pontine gliomas (DIPGs), remain among the deadliest human cancers with a mortality of close to 100% at 5 years and a median survival of one year or less despite aggressive therapies [1]. In the ongoing search for more effective therapies, a more sophisticated understanding of the specific cell biology of these tumors and identifying better therapeutic agents will benefit from high-quality vitro cell lines. Until more recently, few cell lines had been derived from DMGs. Pontine cases were not biopsied because the risks of doing so outweighed the benefit of information gained and neuroimaging sufficed for diagnosis in the vast majority of cases. Autopsies were either not attempted or did not produce viable tissue. However, both the implementation of rapid autopsy techniques and the willingness to perform biopsies for molecular characterization have allowed a number of DIPG cell lines to be developed [2].

In vitro cell cultures have been instrumental in understanding cancer cell biology; however, several issues have provoked questions as to the validity and relevance of some cell culture models. In addition to cross-contamination by cells from other cultures, including from other tumor types and species [3], there has been evidence that cells grown in vitro undergo changes that render them dissimilar to their in vivo counterparts. In gliomas specifically, in vitro cultures have been observed to develop mutations in EGFR not seen in vivo and to lose EGFR amplification, both of which are important biological drivers of IDH-wildtype glioblastomas [4]. An extensive examination of different laboratories’ strains from the same breast carcinoma cell line revealed wide variability in copy-number abnormalities (CNA), the allelic fraction of relevant mutations, and response to chemotherapeutic agents [5]. The clonal evolution of somatic TP53 mutations and MDM2 amplification have been observed in newly created chondrosarcoma cell lines grown as tumor spheres [6]. A temporal analysis of an adipose-derived mesenchymal stromal cell line highlighted genetic divergence, but only with the fifth passage, demonstrating the culture stability of DNA in the first few passages [7]. One study showed the stability of gene expression and copy number profiles in adult glioblastoma cell lines [8]. There has not been a study assessing the stability of DMG’s in cell culture.

Assessing the similarity of in vitro cell cultures of DMG to their in vivo progenitors may give a better understanding of the validity and relevance of these cell lines in screening therapeutic agents and other investigations. The above-noted studies examined the presence of identifying mutations, copy-number abnormalities, or mutagenic effects; yet such approaches have a relatively narrow scope and do not take into account larger-scale changes across the genome that could reflect significant shifts in biology from in vivo to in vitro environments. The genome-wide assessment of DNA methylation patterns by microarray has arguably become the single most powerful tool for diagnosing and subtyping brain tumors [9], creating new diagnostic categories and merging others while revealing some of the limitations of traditional histopathologic diagnosis [10,11]. It interrogates hundreds of thousands of methylation loci throughout the genome to give a highly detailed epigenetic profile for a sample. That pattern can be compared mathematically to those of other known tumors and generate an objective degree of similarity. Because DNA methylation is an important factor in gene expression, such profiles are considered to be highly biologically relevant. While robust enough to reliably identify tumors from formalin-fixed paraffin-embedded (FFPE) and necrotic tissue, it is also sensitive enough to detect the subtle epigenetic effects of aging [12], stress [13], and alcohol consumption [14] in leukocytes. These factors in concert make DNA methylation profiling particularly well suited to assessing the overall stability in the tumor identity of cell cultures relative to their source tumors.

This work describes the results of DNA methylation profiling to examine the impact of in vitro neurosphere culture conditions on cells from H3 K27M-mutant diffuse midline gliomas in children, with the primary aim to assess to what degree the diagnostic profile of the in vitro cells matches that of the original tumor tissue over the course of several passages and describing, if present, what changes develop. Prior works have assessed the similarity of H3 K27M-mutant, patient-derived cell lines to original tumor tissue using DNA methylation profiling, but none have assessed DNA methylation profiling over multiple passages. Castel et al. showed similar methylation profiles by classification algorithm and cluster analysis between eight paired H3 K27M-mutant primary tumors and corresponding adherent monolayer cultures at a single time point [15]. Here, we expand on that work by demonstrating the stability of methylation profiling from three H3 K27M-mutant DIPGs, and enrichment for chromosome arm 1q gain over time.

This study arose following the implementation of neurosphere cell culture at our institution. While many investigators used the cell cultures for their research, occasional concerns were expressed about a tumor in cell culture losing its identity. We therefore decided to investigate whether the identity of a rare cell line was conserved through passages in cell culture. Fresh tumor tissue, available from the operating room, was submitted both for diagnosis and cell culture. We cultured the neurospheres derived from H3 K27M-mutant DIPG’s for up to four passages and submitted the extracted DNA from each passage for methylation profiling. The methylation analysis involved the cluster analysis of the tumors to determine the similarity of the cultured cells with the known methylation profiles of H3 K27M-mutant DIPG’s, but also assessed similarities within cells from the different passages of specific cell lines. This article reports the results of that study.

## 2. Materials and Methods

### 2.1. Overview

All three cell lines of DIPG/DMG were derived from original tumors from patients seen at Children’s Healthcare of Atlanta (Atlanta, GA, USA) and were cultured in the Ian’s Friends Foundation Brain Tumor Biorepository. This study was exempted from review by the Institutional Review Board of Children’s Healthcare of Atlanta because the patients from which the cell lines derive are deceased and not considered human subjects for the purpose of research.

### 2.2. Histology Evaluation and Tumor Specimen Classification

Tissue from all three tumors was submitted fresh from the operating room for frozen section diagnosis. Tumors were submitted for permanent section, and several 1 mm^3^ pieces were submitted in cell culture media (described below) for neurosphere culture. Overnight processing in formalin was performed for diagnosis. Immunohistochemistry was performed on a Leica Bond (Leica Biosystems, Buffalo Grove, IL, USA) and specimens were interpreted by a pediatric neuropathologist.

The three tumors came from H3 K27M-mutant DIPGs in children (Table 1). Specimen 1 was from autopsy and specimens 2 and 3 were from needle core biopsies. All three were diagnosed clinically as diffuse midline glioma, H3 K27M-mutant based on the immunohistochemical expression of H3 K27M mutant protein and absence of H3 K27 trimethylation, in accordance with the 2016 WHO classification of CNS tumors, which was in place at the times of clinical diagnoses [16]. Such cases are termed “diffuse midline glioma, H3 K27-altered” in the 2021 WHO 5th Edition [17]. All three had infiltrating astrocytic tumor cells with irregular, hyperchromatic nuclei, and fibrillar cytoplasmic processes. Specimen one had necrosis and microvascular proliferation, whereas 2 and 3 had only mitotic activity.

### 2.3. In Vitro Cell Culture

**Figure 1:**
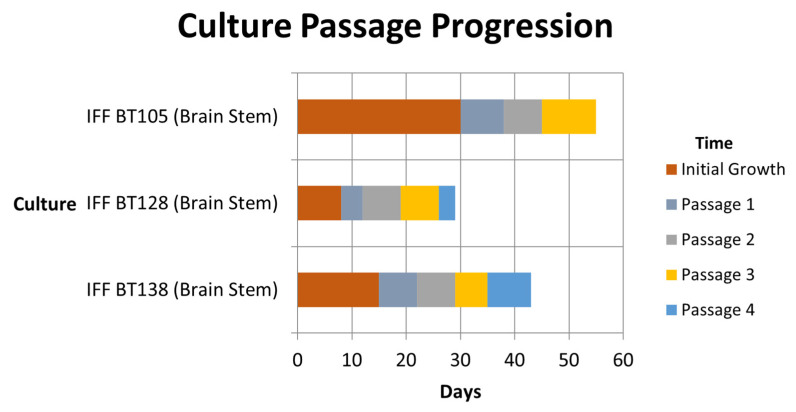
Temporal growth and cell culture passage of three H3 K27M-mutant cell lines.

Fresh, sterile tumor tissue was minced and enzymatically dissociated in 5 mL Accutase (Gibco, Grand Island, NY, USA) per 1 mL tissue at 37 °C for 10–20 min until it consisted of mostly individual cells assessed by inverted microscope. This mixture was diluted with DPBS without Ca^2+^ or Mg^2+^ (Gibco, Paisley, UK) and strained through a 40 micron mesh strainer. The strained mixture was centrifuged at 200× *g* for 10 min. The pellet was re-suspended in RBC lysis buffer (Miltenyi Biotec, Bergisch Gladbach, Germany) for 5 min, centrifuged, washed in serum-free media, and centrifuged again. The cell pellet was re-suspended in supplemented serum-free growth media and incubated at 37 °C in a 5% CO_2_ atmosphere as described in Lin and Monje, 2017 [2]. Each cell culture was dissociated and re-suspended when the neurospheres approached 100 µm in diameter. Passages occurred at differing times among the tumors due to the rapidity with which they grew. A graph demonstrating the passage progression is shown in Figure 1. At each passage, cells were frozen and placed in storage. A total of 98 frozen aliquots of cells were available from these cell cultures to be used by investigators.

Prior to each passage, a cytospin was made on a portion of the cultured cells to assess for identification. The cells were pelleted and both an H&E and an immunohistochemistry stain for the H3 K27M mutant protein was performed. A pediatric neuropathologist confirmed identity as each passage.

### 2.4. DNA Extraction from Brain Tumor Neurospheres

Cultured neurospheres from the frozen cells were collected, pelleted by centrifugation at 200× *g* for 5 min, washed two times in DPBS (-MgCl_2_, -CaCl_2_) and resuspended in 200 µL DPBS (-MgCl_2_, -CaCl_2_) for extraction. The DNA extraction was performed on the Roche MagNA Pure 96 Automated Extractor using the MagNA Pure 96 DNA and Viral NA SV 2.0 Kit with Protocol DNA Cells SV 3.1 (Roche Diagnostics Corporation, Indianapolis, IN, USA). Extracted DNA concentration and purity were measured on NanoDrop-8000 spectrophotometer (Thermo Fisher Scientific, Wilmington, DE, USA).

### 2.5. DNA Extraction of Patient Tumor from FFPE Tissue Blocks

DNA was extracted from FFPE tissue blocks using RecoverAll Total Nucleic Acid Isolation Kit (Invitrogen, Waltham, MA, USA) following the protocol “RecoverAll Multi-Sample RNA/DNA Workflow”. The best block for each case was selected by review of hematoxylin and eosin-stained (H&E) slides by a pediatric neuropathologist. Then, 2 × 10 micron scrolls were cut from the block using a microtome and placed into 1.5 mL microcentrifuge tubes for DNA extraction. Briefly, the scrolls were de-paraffinized using CitriSolv (Decon Laboratories Inc., King of Prussia, PA, USA), the tissue pellet was washed twice with 100% ethanol, and air dried. The tissue was digested with Protease at 55 °C for 1 h followed by an incubation at 90 °C for 1 h. After the Protease digestion was complete, Isolation Additive was added to the sample and then loaded onto a PureLink Mini Column to bind the DNA. The DNA bound to the column was washed with wash buffers included in the RecoverAll kit and then eluted with 30 μL low TE (10 mM Tris pH 8.0, 0.1 mM EDTA) and pre-heated to 95 °C. Extracted DNA concentration and purity were measured on a NanoDrop-8000 spectrophotometer.

### 2.6. Genome-Wide Methylation Profiling

DNA methylation analysis was performed on bisulfite-treated DNA using the Illumina HumanMethylation450 BeadChip (Illumina, San Diego, CA, USA) according to the manufacturer’s instructions. The raw .idat files generated from iScan were processed and analyzed using Bioconductor R (v.4.2.3) package Minfi [18]. All the Illumina array probes were normalized using quantile normalization and corrected for background signal. Samples were then checked for their quality using mean detection *p*-values and probes with a mean detection *p*-value < 0.05 were used for further downstream analysis. Beta values were obtained from the probes that passed the QC as mentioned above. Unsupervised hierarchical clustering was performed on most variable probes using the beta values and heatmap was generated using R package ComplexHeatmap [19]. Red represents hypermethylated probes and blue represents hypomethylated probes in the heatmap.

### 2.7. CNV and tSNE

Raw .idat files from internal cohort cases were run through the DKFZ (German Cancer Research Center, Heidelberg, Germany) methylation classifier for CNS tumors, version 11b6 [9] for generating copy number variation profiles and tSNE (t-distributed stochastic neighbor embedding) plots [20].

## 3. Results

### 3.1. Classifier Scores

The DKFZ brain tumor classifier is a publicly available resource based on a random forest machine-learning algorithm to assess raw methylation data from Illumina Human Methylation 450 BeadChip or EPIC BeadChip arrays. Classifier scores are expressed as a decimal figure ranging from 0.0000 to 1.000, with the latter representing a “perfect” match with a pre-defined diagnostic class based on known reference cases. Each sample is scored for its similarity to each reference diagnostic category, providing a range of entity matches from best to worst. A score of ≥0.90 was established by the DKFZ as a threshold for considering a score diagnostic of an entity [9]. The mean classifier score for the 14 samples was 0.9963, with a range of 0.9817–0.9999, all matching the diagnostic category of diffuse midline glioma, H3 K27M-mutant (Table 1). All other scores for other diagnostic categories were <0.01.

### 3.2. Clustering Analysis

**Figure 2:**
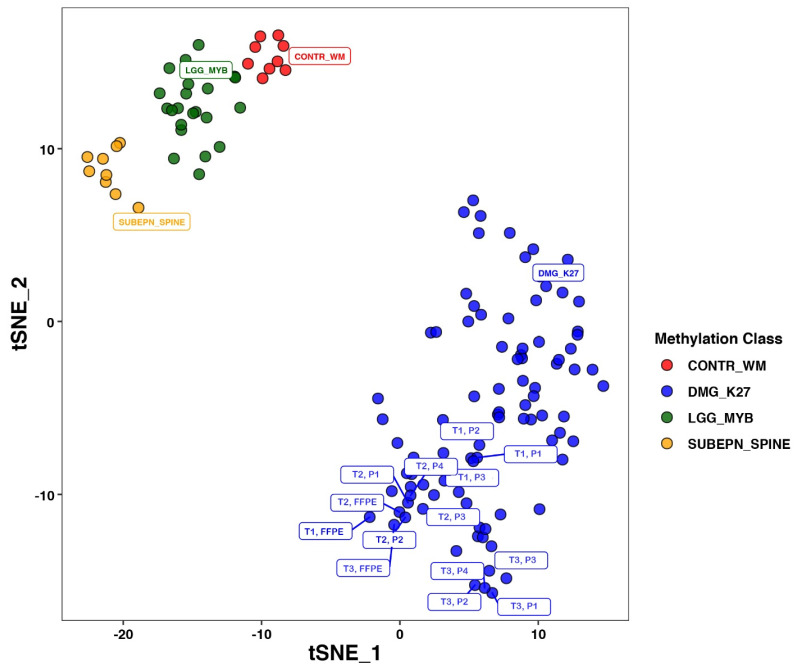
All 14 of the tested samples clustered on the tSNE plot among which the known reference H3 K27M-mutant midline gliomas are separate from the nearest other clusters. T = tumor no., FFPE = biopsy, P = culture passage no.

**Figure 3:**
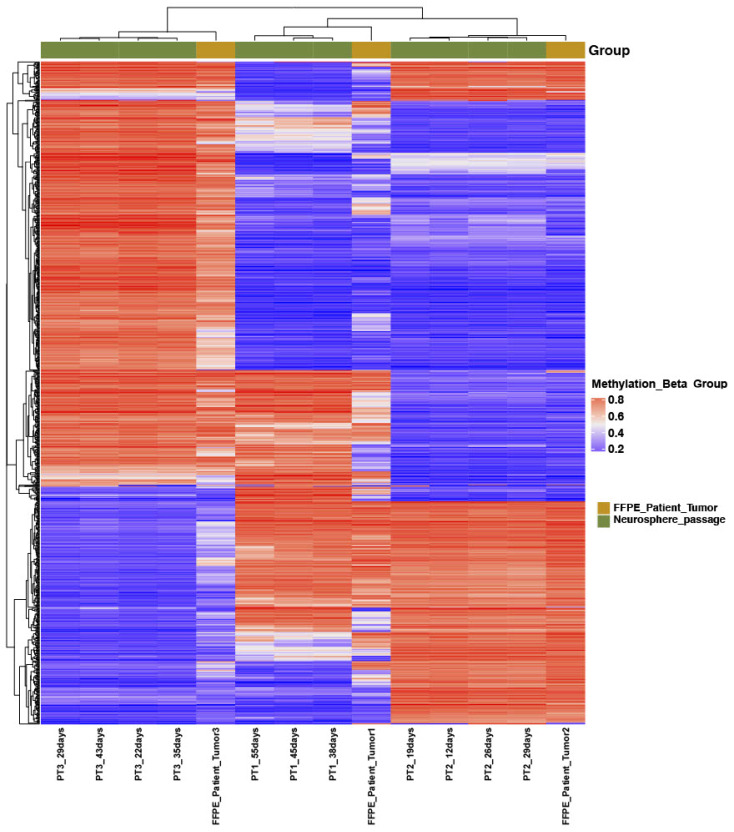
Temporal growth and cell culture passage of three H3 K27M-mutant cell lines identifying similarities within cell cultures for a specific tumor. The red represents hypermethylation and blue represents hypomethylation.

Methylation patterns from all samples clustered as H3 K27M-mutants with a score of 0.99 or higher (Table 1). Multidimensional clustering by t-SNE plot showed all of the samples to be within the reference cluster of known H3 K27M-mutant diffuse midline gliomas and distant from the clusters of other tumor types (Figure 2). Subjected to unsupervised hierarchical clustering, the 14 samples segregated by source case, with the samples from each patient clustering together, indicating that the cell cultures consistently resembled their source tumor more than any of the other samples and there was no overlap between the cultures from different patients (Figure 3). Consistently hyper- and hypomethylated probes across the samples are visualized in Figure 3. Raw data used to derive Figure 3 are presented as Supplementary Materials.

### 3.3. Gain of Chromosome 1q

**Figure 4:**
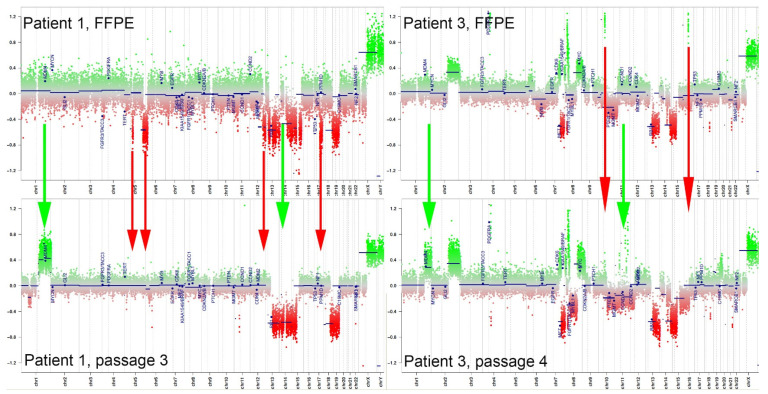
Copy number plots: Initial patient FFPE samples are on top, and the corresponding final culture passages are below. Green arrows indicate copy-number changes gained in culture, and red arrows indicate changes that were lost in culture. Gain of 1q in culture, demonstrated by the green arrow on the left side of each image, was the only change in common between the two. Tumor 2 had gain of 1q in all tested samples.

Although not directly measured, a copy number can be inferred from DNA methylation array data with an accuracy that falls short of—but is roughly comparable to—that of SNP-based copy number microarrays [21]. Case 1 showed deletions of 5q, 12q, 13q, 14q, 15q, 17p (*TP53*), and 18q in the original biopsy tissue and a gain of 1q (q21.1–q44) with whole chromosome losses of 13, 14, and 18 in the final passage of the cultured cells. There were transient short-segment losses on 2q and 6p that resolved on the final passage (Figure 4). Case 2 initially had a gain of most of the long arm of chromosome 1 (1q21.1–q44), which remained as the sole copy-number abnormality in the cultured cells throughout. The biopsy tissue from case 3 showed a short-segment amplification of *PDGFRA*, gains of 2q and 8p, and losses of 10q (*PTEN*), 13p (*RB1*), and 14q. 7q was mostly lost but also had gains of segments containing *EGFR* and *BRAF*. A few other small amplifications were noted on 8p, 10p, and 15p, none of which contained obvious growth-driving genes. As with tumor 1, cultured cells from tumor 3 also showed an enrichment of cells with a gain of 1q (q31.1–q44). Other changes included a new low-level loss of chromosome 8 spanning the centromere and leaving the previously noted gains, low-level monosomy of 11 and 15, and gain of most of 19p intact. The small amplifications on 10q and 15p did not persist to the last passage, and two others were gained on 11q, also without obviously relevant genes.

## 4. Discussion

Inasmuch as DNA methylation profiles represent the overall identities of different tumor types, these data suggest that H3 K27M-mutant diffuse midline gliomas are fundamentally stable in neurosphere culture conditions, at least through the initial several passages. Nevertheless, there were changes observed in the cultured cells that require further examination.

The modest shifts in methylation profiles from the original tumor tissue to the cultures are most likely due to two factors. The FFPE samples are by their nature contaminated by endothelial cells, glia, and other native cells that were not present in the cultures, thereby contaminating the data with their own different profiles, albeit at a low level. Chromosomal copy-number changes may have had some impact on the methylation profiles, but to a smaller degree than contamination because they were inconsistent between tumors. The fact that the clinical samples were embedded in FFPE and the cultures were not is unlikely to have had any major effect on the methylation profile, but the minor impact seen in our study could be expected [22,23]. Additional studies could be performed by isolating the tumor cells from the background endothelial and glial cells and determining whether the phenotype of isolated tumor cells more closely resembles the neurospheres.

H3 K27M-mutant DIPGs do not have a consistent copy-number profile, yet there are a few changes that are common in subsets of them. In terms of large chromosomal changes, the most common gain is that of 1q, which is present in 23–69% of DIPG cases [24,25,26] and 64% (18 of 28) in one series restricted to H3 K27M-mutant cases [27]. After just the first passage, all three cell lines had an enriched population of cells with 1q chromosomal gain, even though tumors 1 and 3 did not show evidence of that originally. We postulate that there may have been clones containing a gain of chromosome 1q in tumors 1 and 3 that provided a growth advantage, and therefore expanded out of proportion to cells without 1q in subsequent passages.

Gains of 1q are common in the cancers of many organs and have been associated with a worse prognosis in Wilms tumor [28], multiple myeloma [29], and papillary thyroid carcinoma [30], among others. This association has also been observed in CNS tumors, with the gain of 1q associated with worse prognosis in ependymomas [31], atypical meningiomas [32], diffuse leptomeningeal glioneuronal tumors [33], and pediatric diffuse gliomas [34]. If 1q gain promotes the tumor growth, it may be a strong selective pressure for any of those cells to dominate the culture population. One series observed that the increased levels of the mutant H3F3A K27M allele, which is located on 1q, are associated with worse survival [35]. In contrast, another series observed that 1q gains were more common H3 K27M-mutant DIPGs with HIST1H3B (H3.1) mutations, which have a slightly longer median survival, versus those with H3F3A (H3.3) gene mutations [27]. A greater investigation is required to assess the significance of 1q gains in DMG biology and clinical behavior.

Taken together, these results support the in vitro cell culture of H3 K27M-mutant DMGs as a method for propagating tumor cells while retaining their essential identity as representative of those found in the original tumor during the first few passages. Because in vitro cultured cells are convenient for investigating tumor cell biology and amenable to the high-throughput assays of sensitivity to potential therapeutic agents, it is reassuring that the methylation profiles are stable at least for several passages over several weeks to months. One potential limitation of our study is that we did not perform these analyses with a greater number of cultures or for longer periods of time. However, our goal was to determine whether identity was achieved in these rare cells lines, which are typically available from small-cell culture biorepositories rather than large-cell culture laboratories providing cell lines commercially. From these three samples, passaged between 3 and 4 times, we achieved 98 aliquots to be used by investigators, who can be confident in the identity of the cell culture material they receive. These and similarly cultivated and validated cell lines promise to provide an invaluable, biologically relevant resource for DMG/DIPG investigators.

## 5. Conclusions

This study demonstrates that a rare pediatric brain tumor, H3 K27M-mutant diffuse midline glioma, can be cultured in vitro for up to four passages and maintain its identity with the same methylation profile as the primary tumor. The practical application of this information is to reinforce that we did not identify tumor drift, which is well known to occur in the cell culture of some tumors. Therefore, these cells can be used for experiments to assess biology or potential therapy to treat this malignant tumor, Although not affecting the identity of the cultured cells, there were slight differences in methylation profiles between the cultured tumor cell DNA and the DNA extracted from the formalin-fixed paraffin-embedded diagnostic material, which contains tumor cells in addition to background cerebral matrix and endothelial cells. The literature supports that the difference in methylation is due to the presence of non-tumor cells in the paraffin-embedded diagnostic tissue, but further studies could be performed to separate tumor cells from other cellular components in the formalin-fixed paraffin-embedded tissue to confirm this hypothesis. Ian’s Friends Foundation, which supports the brain tumor biorepository that produces these cells lines to investigators free of charge, and also supported this research to assess cell identity, states their vision simply. It is to create a world free of pediatric brain tumors. We hope that this biorepository and this article will help move us all toward this vision.

## Figures and Tables

**Table 1:** Summary of patients and samples with classification as diffuse midline glioma, H3 K27M-mutant (DMG H3 K27M) and DKFZ classifier score out of potential maximum 1.000.

Age */Sex	Sample No.	Sample ID	Classification	Score
5F	1	IFF BT105 FFPE patient tumor	DMG H3 K27M	0.9962
	2	IFF BT105 F1p1d38 (passage 1)	DMG H3 K27M	0.9931
	3	IFF BT105 F1p2d45 (passage 2)	DMG H3 K27M	0.9963
	4	IFF BT105 F1p3d55 (passage 3)	DMG H3 K27M	0.9963
2M	5	IFF BT128 FFPE patient tumor	DMG H3 K27M	0.9817
	6	IFF BT128 F1p1d12 (passage 1)	DMG H3 K27M	0.9973
	7	IFF BT128 F1p2d19 (passage 2)	DMG H3 K27M	0.9967
	8	IFF BT128 F1p3d26 (passage 3)	DMG H3 K27M	0.9959
	9	IFF BT128 F1p4d29 (passage 4)	DMG H3 K27M	0.9958
8F	10	IFF BT138 FFPE patient tumor	DMG H3 K27M	0.9999
	11	IFF BT138 F1p1d22 (passage 1)	DMG H3 K27M	0.9998
	12	IFF BT138 F1p2d29 (passage 2)	DMG H3 K27M	0.9998
	13	IFF BT138 F1p3d35 (passage 3)	DMG H3 K27M	0.9997
	14	IFF BT138 F1p4d43 (passage 4)	DMG H3 K27M	0.9994

* Age represented in years

## Data Availability

The original contributions presented in the study are included in the article/Supplementary Materials, further inquiries can be directed to the corresponding author.

## Supplementary Materials

The following supporting information can be downloaded at: https://www.mdpi.com/article/10.3390/children11040492/s1.
